# Integrating Genome-Scale Metabolic Modeling with Machine Learning Improves Gene Essentiality Prediction in Triple-Negative Breast Cancer

**DOI:** 10.3390/ijms27115059

**Published:** 2026-06-03

**Authors:** Bo Kyung Kim, Changdai Gu, Mohamed El-Agamy Farh, Jae Yong Ryu

**Affiliations:** 1 Artificial Intelligence Laboratory, Oncocross Co., Ltd., 7, Beobwon-ro 11-gil, Songpa-gu, Seoul 05836, Republic of Korea; 2 Department of Artificial Intelligence, School of Computing, Yonsei University, 50 Yonsei-ro, Seodaemun-gu, Seoul 03722, Republic of Korea; 3 Medical Research Center, College of Medicine, Yonsei University, 50 Yonsei-ro, Seodaemun-gu, Seoul 03722, Republic of Korea; 4 AI-Bio Convergence Research Institute, Soongsil University, 369 Sangdo-ro, Dongjak-gu, Seoul 06978, Republic of Korea; 5 School of Systems Biomedical Science, Soongsil University, 369 Sangdo-ro, Dongjak-gu, Seoul 06978, Republic of Korea

**Keywords:** breast cancer, genome-scale metabolic model, machine learning, synthetic lethality

## Abstract

Triple-negative breast cancer (TNBC) poses a significant therapeutic challenge owing to its aggressiveness and limited treatment options. Here, we integrated genome-scale metabolic modeling with machine learning to improve gene essentiality prediction and identify candidate therapeutic targets for TNBC. Cell-line-specific genome-scale metabolic models were reconstructed for 50 breast cancer cell lines using RNA-sequencing from Cancer Dependency Map (DepMap). Metabolic reaction flux distributions derived from minimization of metabolic adjustment (MOMA) were used as features to train a random forest classifier, with DepMap gene dependency scores as ground truth labels. This integrative approach outperformed the MOMA alone for gene essentiality prediction, increasing sensitivity from 0.37 to 0.55. The model identified 57 TNBC-specific essential genes, including Enolase 1 (*ENO1*), that were missed by MOMA-based prediction. Furthermore, 30 synthetic lethal partners of succinate dehydrogenase subunit A (*SDHA*) were predicted in TNBC cell lines. This framework demonstrates the utility of combining metabolic modeling with machine learning for identifying context-specific cancer vulnerabilities.

## 1. Introduction

Breast cancer is among the most prevalent malignant tumors and a leading cause of cancer mortality in women, with approximately 2.3 million new cases reported, along with more than 665,000 deaths in 2022 [1,2]. It is a heterogeneous disease classified into four subtypes based on the gene expression status of the estrogen receptor (ER), progesterone receptor (PR), and human epidermal growth factor receptor 2 (HER2): luminal A (ER+, PR+, HER2−), luminal B (ER+, PR+, HER2+), HER2-positive (ER−, PR−, HER2+), and triple-negative breast cancer (TNBC; ER−, PR−, HER2−) [3,4,5,6,7]. Among these, TNBC is the most aggressive breast cancer subtype, characterized by high risk of recurrence and metastasis, poor prognosis, and lack of targetable receptor, which render it unresponsive to endocrine or anti-HER2 therapies [8,9,10].

Metabolic reprogramming is a hallmark of cancer that enables cells to survive and proliferate under nutrient-limited conditions [11]. A well-characterized example is the Warburg effect, in which cancer cells preferentially utilize aerobic glycolysis despite the availability of oxygen [12,13]. Compared with other breast cancer subtypes, TNBC exhibits heightened dependence on glycolysis and undergoes metabolic reprogramming involving oxidative phosphorylation, amino acid metabolism, and lipid metabolism to meet energetic and biosynthetic demands of cells [14]. These altered metabolic pathways represent potential therapeutic targets for TNBC treatment.

Numerous studies have elucidated cancer-specific metabolic pathways and leveraged this knowledge to identify potential anticancer targets whose inhibition impairs tumor growth and survival [15,16,17,18]. Targeting the altered metabolism of cancer cells is a promising therapeutic strategy; however, achieving selectivity over normal cells requires a comprehensive understanding of the underlying metabolic network. Genome-scale metabolic models (GEMs) provide a computational framework for this purpose [19,20]. GEMs are stoichiometric computational reconstructions that integrate all known metabolic genes, enzymes, and reactions within a cell, enabling simulation of metabolic flux distributions under specific genetic and environmental condition. By predicting metabolic consequences of genetic perturbations, GEMs can identify cancer cell-specific metabolic vulnerabilities and potential therapeutic targets [21,22,23,24,25,26].

Previous studies have demonstrated the utility flux balance analysis (FBA) and MOMA-derived GEM-based approach for predicting anticancer targets through metabolic modeling [27,28]. While traditional FBA assumes steady-state network optimization for maximum biomass growth, context-specific extraction tools like the task-driven Integrative Network Inference for Tissues (tINIT) algorithm are required to tailor generic human templates into cell-specific models by integrating transcriptomic data. To simulate genetic knockouts within these networks, the MOMA algorithm relaxes FBA’s optimal-growth assumption. MOMA utilizes quadratic programming to identify a mutant flux distribution that minimizes the Euclidean distance from the unperturbed wild-type flux state, capturing the immediate, non-evolutionary metabolic response to a reaction perturbation. However, constraint-based simulation methods, including FBA and MOMA, remain limited in their ability to predict gene essentiality in eukaryotic cells [29]. Therefore, new methods are required to improve the accuracy of essential gene predictions and identify potent anticancer targets.

The Cancer Dependency Map (DepMap) project has systematically catalogued gene essentiality across over 1000 cell lines through genome-wide CRISPR-Cas9 screens, providing quantitative gene dependency scores (GDSs) that distinguish common essential genes from context-specific vulnerabilities [30,31]. This large-scale dataset serves as an excellent benchmark for evaluating computational approaches to gene essentiality prediction in cancer.

**Figure 1:**
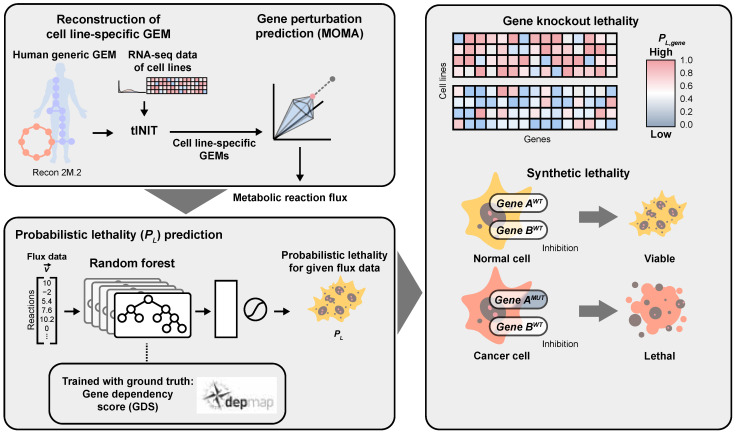
Overall study scheme. RNA-sequencing (seq) data of 50 breast cancer cell lines (8 luminal A, 7 luminal B, 10 HER2-positive, and 25 TNBC) from the DepMap portal were collected. Cell-line-specific genome-scale metabolic models (GEMs) were reconstructed using human generic GEM Recon 2M.2, applying the RNA-seq data using the tINIT algorithm. Using MOMA, genetically perturbed metabolic flux was predicted for the reconstructed GEMs. A random forest (RF) model was used to predict the probabilistic lethality (*P_L_*) of gene perturbation (ranging from 0 to 1, where 1 is lethal) for the flux data. The RF model was trained with the gene dependency score (GDS) of the DepMap portal, ahead of prediction. Using the MOMA–RF-based approach, the lethality of gene knockouts was predicted, which indicates essential genes. Synthetic lethality of gene pairs was predicted, where genes are non-lethal individually but lethal when disrupted simultaneously.

In this study, we integrated genome-scale modeling and GDSs with machine learning to improve gene essentiality prediction in breast cancer (Figure 1). Cell-line-specific GEMs were reconstructed for 50 breast cancer cell lines using the tINIT algorithm and Recon 2M.2, and MOMA-derived flux distributions were combined with GDSs from DepMap as inputs to train and evaluate multiple machine learning classifiers. Using this integrative framework, we identified TNBC-specific essential genes and synthetic lethal pairs that may serve as candidate therapeutic targets for this aggressive breast cancer subtype.

## 2. Results and Discussion

### 2.1. Reconstruction of 50 Breast Cancer Cell Line-Specific Genome-Scale Metabolic Models

**Figure 2:**
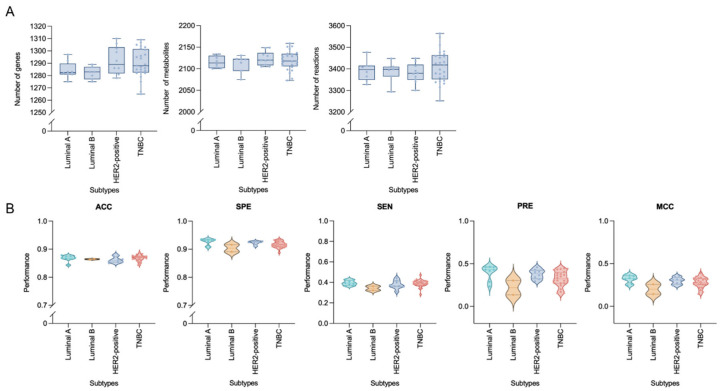
Reconstruction of breast cancer cell line-specific genome-scale metabolic models (GEMs). (**A**) Numbers of genes, metabolites, and reactions in 50 cell-line-specific GEMs corresponding to four breast cancer subtypes: Luminal A (8 cell-line-specific GEMs), luminal B (7 cell-line-specific GEMs), HER2-positive (10 cell-line-specific GEMs), and TNBC (25 cell-line-specific GEMs). Each point represents the number in each cell line of each breast cancer subtype. (**B**) Performance of the MOMA model. Violin plot of model performance for each cell line of the four breast cancer subtypes. Each point in the violin plot represents the performance of an individual cell line. ACC, accuracy; SPE, specificity; SEN, sensitivity; PRE, precision; MCC, Matthews correlation coefficient. Green, luminal A breast cancer; orange, luminal B breast cancer; blue, HER2-positive breast cancer; red, TNBC.

Cell-line-specific GEMs were reconstructed for 50 breast cancer cell lines spanning four molecular subtypes, including TNBC, by integrating RNA-seq expression with generic human GEM Recon 2M.2 using the tINIT algorithm (see Section 3). Recon 2M.2 was selected as a reference network based on its superior gene essentiality prediction compared with Recon3D and Human 1 [25,26,32,33,34]. This choice is supported by comparative benchmarks indicating that the expanded structural architectures and inflated gene-protein-reaction (GPR) annotations in later iterations often introduce unconstrained parallel bypasses that can compromise the accuracy of phenotype-driven essentiality predictions [33,34]. The reconstructed GEMs exhibited subtype-specific features in network size (Figure 2A). The GEMs for HER2-positive breast cancer cell lines contained the highest average number of genes (1292) and metabolites (2124), whereas the GEMs for TNBC contained the highest number of reactions (3412), consistent with extensive metabolic reprogramming characteristic of this subtype.

### 2.2. Gene Essentiality Prediction Using Machine Learning Models

The baseline performance of MOMA alone for gene essentiality prediction was first evaluated for all 50 cell lines (Figure 2B). MOMA achieved an average accuracy (ACC) of 0.86 and specificity (SPE) of 0.92, sensitivity (SEN) of 0.37, precision (PRE) of 0.33, and Matthews correlation coefficient (MCC) of 0.27. The high accuracy and specificity are largely attributed to strong class imbalance in the dataset. The low sensitivity means that MOMA correctly identified 37% of experimentally validated essential genes, with the majority of true dependencies missed. These results established the need for an improved prediction framework that can enhance sensitivity without compromising specificity. While integrating sequence-centric models like AlphaGenome [35] represents a promising future direction for linking genomic alterations to flux, this study focuses specifically on addressing the sensitivity limitations of mechanistic metabolic models.

**Figure 3:**
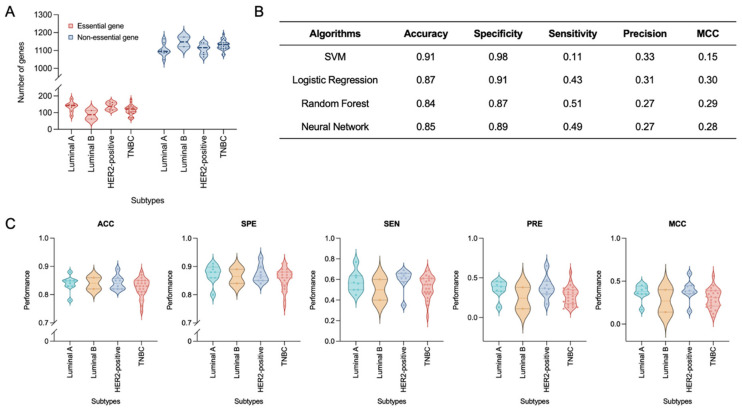
Performance evaluation of various machine learning approaches for gene essentiality prediction in different breast cancer subtypes. (**A**) Distribution analysis of predicted essential and non-essential genes in different breast cancer subtypes revealing a class imbalance in gene essentiality data. The data points of the violin plots show the number of essential (red) and non-essential (blue) genes in each subtype. (**B**) Comparative assessment of the efficiency of different machine learning methods to handle data imbalance. Random under-sampling shows optimal performance when assessing the performances using the MDA-MB-231 cell line as a representative TNBC cell line. (**C**) Comprehensive performance metrics of the optimized random forest model for various breast cancer subtypes. ACC, accuracy; SPE, specificity; SEN, sensitivity; PRE, precision; MCC, Matthews correlation coefficient. Green, luminal A breast cancer; orange, luminal B breast cancer; blue, HER2-positive breast cancer; red, TNBC.

To improve upon MOMA baseline, four machine learning classifiers, support vector machine (SVM), logistic regression (LR), random forest (RF), and neural network (NN), were trained using MOMA-derived knock-out flux vectors as features and binarized GDS labels as prediction targets (Figure 3). Model development was processed in three stages using MDA-MB-231-specific GEM, a TNBC cell line. First, three class-sampling strategies were compared to address imbalance determined between essential and non-essential genes (Figure 3A). Among the three strategies, over-sampling achieved the highest accuracy and specificity, but the lowest sensitivity and MCC. Both random under-sampling and combined Synthetic Minority Oversampling Technique combined with edited nearest (SMOTE-ENN) achieved comparable median sensitivity and MCC; however, random under-sampling produced substantially more consistent performance across cell lines, with narrower inter-quantile range across all metrics (Supplementary Figure S1A). Random under-sampling was, therefore, selected for all subsequent model training. Second, the four classifiers were compared on the MDA-MB-231-specific GEM (Figure 3B). All four models achieved similar accuracy, but RF and NN exhibited substantially higher sensitivity. Third, to distinguish between RF and NN, both classifiers were trained and evaluated across all 25 TNBC cell-line-specific GEMs (Supplementary Figure S1B). RF demonstrated more consistent performance with narrower variance across different cell lines compared with NN, and was, therefore, selected as the final classifier.

RF-based models were trained for all 50 breast cancer cell line GEMs across the four subtypes (Figure 3C). The RF models showed a mean accuracy of 0.84, specificity of 0.87, sensitivity of 0.55, precision of 0.31, and MCC of 0.33. Compared with MOMA alone, the MOMA–RF approach improved sensitivity by 49% in relative terms (from 0.37 to 0.55) and MCC by 22% (from 0.27 to 0.33) (Figure 2B and Figure 3C, Supplementary Figures S2 and S3, and Supplementary Table S5), indicating that larger proportions of experimentally validated essential genes were correctly identified This is accompanied with slightly lower accuracy and specificity, reflecting the expected trade-off when the classifier shifts towards higher recall in an imbalanced dataset. Importantly, MCC, which accounts for all four entities of the confusion matrix and is more robust to class imbalance, improved constantly, indicating that the gain of sensitivity is not simply an artifact of a more robust liberal classification threshold. Performance trended positively in all four breast cancer subtypes (Figure 3C, Supplementary Figures S2 and S3), with statistical significance gained in TNBC and HER2-positive cell lines, and a non-significant trend in luminal A and luminal B subtypes (Supplementary Table S5) likely reflecting limited sample size. These results suggest that the integrative approach generalized beyond TNBC, along with other cancer lineages. Furthermore, future iterations utilizing updated consensus models such as Human2 [36] or graph-based architectures like FlowGAT [28] may further enhance prediction accuracy. While tools like FlowGAT leverage graph attention networks to learn natively from network topology, our current approach focuses explicitly on mechanistic flux redistribution profiles. We intentionally selected an RF architecture for its robustness and high interpretability in handling these tabular flux features. Our framework demonstrates that even when built upon standard structural templates, the targeted integration of machine learning successfully recovers critical true essential genes missed by traditional constraint-based flux methods.

### 2.3. Prediction of TNBC-Specific Essential Genes

**Figure 4:**
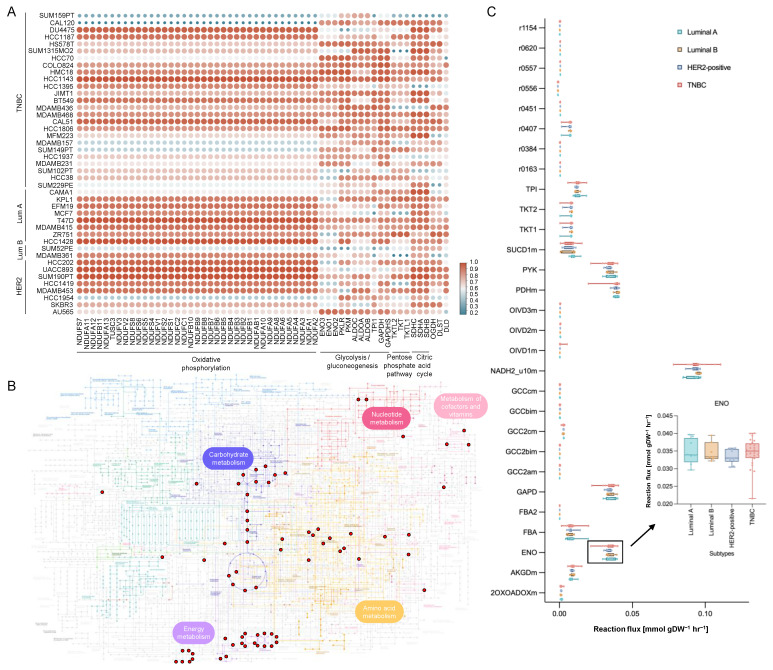
Machine-learning-based identification and characterization of TNBC-specific essential genes. (**A**) Comparative analysis of the gene essentiality probability across breast cancer subtypes for 57 TNBC-specific essential genes identified via machine learning prediction (probability > 0.5). These genes were selected from 298 candidates initially predicted as non-essential by MOMA and essential by the machine learning model. Data points are categorized by metabolic pathways, with essentiality indicated by color intensity (red, essential; blue, non-essential). (**B**) Pathway enrichment analysis of TNBC-specific essential genes reveals predominant involvement in carbohydrate, amino acid, and energy metabolism pathways. This distribution reflects the characteristic of metabolic reprogramming in TNBC. (**C**) Quantitative comparison of the metabolic flux distribution for reactions associated with the predicted essential genes across breast cancer subtypes; enolase (*ENO*) family genes (*ENO1*, *ENO2*, and *ENO3*) exhibited distinct subtype-specific metabolic activity. For detailed reaction name and related genes, see Supplementary Table S6. Green, luminal A breast cancer; orange, luminal B breast cancer; blue, HER2-positive breast cancer; red, TNBC.

To identify TNBC-specific essential genes, RF model predictions were compared with MOMA-based ones across all four cancer subtypes (Supplementary Figure S4). A total of 298 genes were classified as non-essential by MOMA but essential by the RF models. Of these, 57 were predicted as essential (*P_L_* > 0.5) for the majority of TNBC cell lines while showing lower essentiality probabilities in luminal A, luminal B, and HER2-positive cell lines, indicating subtype-specific dependency (Figure 4A). Importantly, these 57 genes are verified to be among the true essential genes based on the experimental DepMap dataset, demonstrating that MOMA–RF successfully captures biological ground truth. This correction occurs because standalone MOMA operates strictly on structural network stoichiometry; it often identifies mathematically redundant, alternative metabolic pathways to bypass a simulated knockout, resulting in false negatives. Conversely, MOMA–RF integrates cell-line-specific transcriptomic constraints via the random forest layer, allowing it to recognize when these theoretical metabolic bypasses are actually transcriptionally silent or restricted in true TNBC lineages, thereby correctly identifying context-specific essential genes. These 57 TNBC-specific essential genes were predominately involved in carbohydrate, amino acid, and energy metabolism (Figure 4B), consistent with glycolytic and biosynthetic characteristics of TNBC. Among the 57 TNBC-specific essential genes, *ENO1*, *ENO2*, and *ENO3*—members of the enolase (*ENO*) family which catalyzes the conversion of 2-phosphoglycerate to phosphoenolpyruvate in the penultimate step of glycolysis —were of particular interest. All *ENO* genes exhibited higher mean expression (Supplementary Figure S6) and elevated flux values (Figure 4C) in TNBC cell lines than those in other breast cancer subtypes. Within the ENO family, *ENO1* showed the strongest TNBC-specific dependency, as reflected by the most distinct separation in GDSs between TNBC and other subtypes (Supplementary Figure S5), the highest expression among the three isoforms, and the largest flux contribution in TNBC cell lines (Figure 4C and Supplementary Figure S6). These computational predictions are supported by existing experimental evidence. *ENO1* inhibition has been shown to suppress migration, proliferation, and invasion in breast cancer cells [37] and to reduce colony formation and tumor growth while promoting cell death specifically in TNBC cell lines [38]. These findings support the biological plausibility of *ENO1* as a TNBC-specific vulnerability and suggest that the broader set of 57 genes identified by the MOMA–RF framework may contain additional candidates warranting experimental validation. While this machine learning framework was primarily validated at the cell-line level due to the absolute requirement of experimental ground-truth essentiality screens for model evaluation, the downstream TNBC-specific metabolic targets identified here represent highly promising candidates for direct translation. Future studies validating these core vulnerabilities in real patient cohorts, such as The Cancer Genome Atlas (TCGA) dataset, will further confirm their clinical utility as diagnostic biomarkers and therapeutic targets.

### 2.4. Prediction of Synthetic Lethal Gene Combinations in Triple-Negative Breast Cancer

The MOMA–RF was next applied to predict the synthetic lethal gene pairs in TNBC. SDHA, which encodes the catalytic subunit of succinate dehydrogenase (mitochondrial complex II), was selected as the benchmark anchor gene based on its vital role in central energy metabolism and its mutation in approximately 3% of all breast cancer patients [39]. While standard targeted clinical regimens for TNBC predominantly focus on DNA damage repair (DDR) pathways via *BRCA1/2* status, Homologous Recombination Deficiency (HRD) scores, or Poly(ADP-Ribose) Polymerase (*PARP*) inhibitors, we intentionally selected SDHA to investigate non-canonical metabolic vulnerabilities. Because *SDHA* functionally couples the tricarboxylic acid (TCA) cycle and the mitochondrial electron transport chain, it serves as a critical central hub for evaluating global flux redistribution, offering alternative metabolic therapeutic targets that complement established genomic paradigms. For this, SDHA knockout was simulated by constraining the SUCD1m reaction to zero flux in each cell-line-specific GEM. MOMA predicted that SDHA single-knockout did not reduce growth rates below the 5% lethality threshold in any TNBC cell line, indicating that SDHA loss alone is tolerated—a prerequisite for synthetic lethality screening, in which the anchor gene must be non-lethal individually. In double-knockout simulations using the MOMA alone, only two genes, fumarate hydratase (FH) and *SLC25A3*, exhibited a synthetic lethal with SDHA, highlighting the limited capacity of constraint-based simulation to detect combinatorial vulnerabilities. While *FH* has been documented to function as an upregulated metabolic oncogene that promotes breast cancer tumorigenesis [40], its linear metabolic proximity to *SDHA* in the TCA cycle ensures that their concurrent ablation creates a non-bypassable structural constraint in steady-state flux networks.

**Figure 5:**
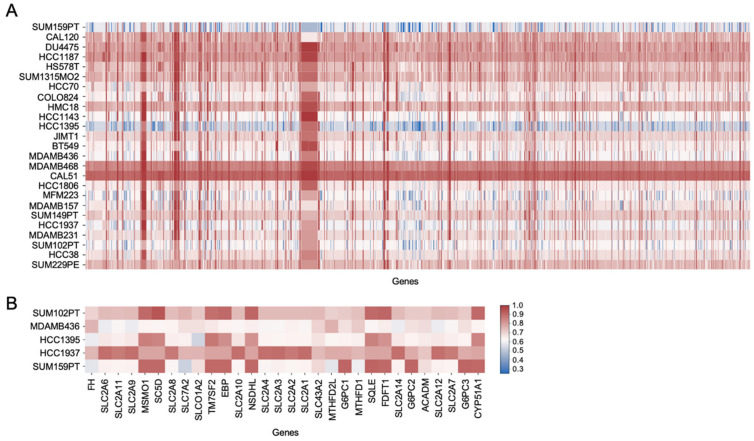
Prediction of synthetic lethal gene combinations with *SDHA* in TNBC via machine-learning-based synergy analysis. (**A**) *Syn_L_*, Synergy score evaluation following SUCD1m reaction removal across TNBC cell lines. The heatmap shows the synthetic lethality predictions based on the following criteria: *P_L,i_* ≤ 0.5 for all genes *i*, and *P_L,i,j_* > 0.5 for double-knockout of genes *i* and *j* in TNBC cell lines, where *P_L_* indicates the probabilistic lethality measured using cell-line-specific machine learning models and MOMA-based gene perturbation flux results. Color intensity indicates the degree of essentiality (red, essential; blue, non-essential). (**B**) Quantitative synergy scores of the top 30 candidate genes exhibiting synthetic lethal relationships with *SDHA*, including *FH*, *SLC2A* family members, and metabolic enzymes. This expanded prediction highlights the superiority of the machine learning approach over MOMA-based analysis, which only identified *FH* and *SLC25A3* as synthetic lethal combinations.

To identify additional synthetic lethal gene pairs beyond the two detected by MOMA alone, the trained RF models were used to predict the probabilistic lethality, *P_L,SDHA,i_*, of pairwise SDHA + gene *i* double-knockouts across TNBC cell-line-specific GEMs. Because SDHA single-knockout was predicted as lethal (*P_L_* > 0.5) in 20 of 25 TNBC cell lines, the synthetic lethality analysis was restricted to the five cell lines in which SDHA loss alone was non-lethal (*P_L_* < 0.5), consistent with the prerequisite that the anchor gene must be individually tolerated. Candidate synthetic lethal partners were selected using two criteria: (i) single-knockout of gene *i* was non-lethal (*P_L,__i_* ≤ 0.5), and (ii) the SDHA + gene *i* double-knockout was lethal (*P_L,SDHA,i_* > 0.5). The synergy score of lethality, *Syn_L_*, was calculated using the Bliss independence model to assess the degree of the double-knockout lethality (Figure 5A). This analysis identified 30 genes exhibiting synthetic lethality with *SDHA*: *FH*, *SLC2A6*, *SLC2A11*, *SLC2A9*, *MSMO1*, *SC5D*, *SLC2A8*, *SLC7A2*, *SLCO1A2*, *TM7SF2*, *EBP*, *SLC2A10*, *NSDHL*, *SLC2A4*, *SLC2A3*, *SLC2A2*, *SLC2A1*, *SLC43A2*, *MTHFD2L*, *G6PC1*, *MTHFD1*, *SQLE*, *FDFT1*, *SLC2A14*, *G6PC2*, *ACADM*, *SLC2A12*, *SLC2A7*, *G6PC3*, and *CYP51A1* (Figure 5B and Supplementary Figure S7).

These predicted targets clustered tightly into functional pathways previously established to compensate for mitochondrial Complex II failure in alternative respiratory-deficient tumors. The largest functional cohort comprised glucose transporters and glycolytic regulators, including twelve members of the solute carrier family 2 (*SLC2A1–SLC2A4*, *SLC2A6–SLC2A12*, *SLC2A14*) and glucose-6-phosphatase catalytic subunits (*G6PC1–G6PC3*), reflecting an absolute network dependency on the Warburg shift and accelerated glucose influx to bypass a broken TCA cycle [41,42]. Additionally, MOMA–RF mapped a severe co-dependency within the cholesterol and sterol biosynthetic pathways (*SQLE*, *FDFT1*, *MSMO1*, *SC5D*, *TM7SF2*, *EBP*, *NSDHL*), a known metabolic vulnerability driven by altered mitochondrial redox and lipid homeostasis [43]. Other high-priority candidates included folate pathway enzymes (*MTHFD1*, *MTHFD2L*), fatty acid oxidation (*ACADM*), and solute transporters (*SLC7A2*, *SLCO1A2*, *SLC43A2*). These newly identified in silico targets provide a mechanistically reasonable landscape of metabolic vulnerabilities specific to TNBC cell lines, serving as prioritized candidates for future experimental validation. All 30 gene pairs showed positive synergy scores, confirming that the combined lethality exceeded the expected additive effect of individual knockouts (Figure 5B). Conversely, *SLC25A3*—though flagged as a structural vulnerability by MOMA alone—was excluded by MOMA–RF (*P_L_* < 0.5) because its contextual transcriptomic features do not support a high probability of double-knockout lethality in these lineages. Notably, several of these prioritized targets are highly druggable, including the glycolytic network (via selective *GLUT1* inhibitors like BAY-876) [44] and the sterol pathway (via SQLE inhibitors) [45].

Crucially, the identification of these specific candidates by MOMA–RF represents a direct, automated reproduction of previously reported metabolic synthetic lethal interactions, demonstrating the methodological validity of our framework. For instance, the synergistic co-dependency linking succinate dehydrogenase (SDH) failure to accelerated glycolytic reliance (SLC2A family) and alternative TCA cycle disruptions (FH) precisely reproduces established therapeutic lethal axes validated in independent respiratory-deficient tumor models [40,41,42]. Similarly, the captured vulnerabilities within downstream sterol pathways mirror documented synthetic lethal phenotypes driven by mitochondrial redox imbalances [43]. By successfully recapturing these literature-validated metabolic pairs, the MOMA–RF framework demonstrates clear structural rigidity, proving it can isolate genuine biological dependencies from computational simulation space without accumulating high false-positive rates. From a safety perspective, our in silico single-knockout simulations confirmed that individual ablation of these 30 candidate genes does not compromise baseline biomass flux, strictly satisfying the operational definition of synthetic lethality. However, thoroughly characterizing potential toxicity across the entire physiological spectrum of healthy tissues demands exhaustive empirical evaluation across a broad panel of normal cell lines, an essential objective reserved for further prospective studies. Hence, these genes represent potential drug targets for *SDHA*-mutated TNBC cases.

## 3. Materials and Methods

### 3.1. Dataset

RNA-seq expression data and CRISPR-Cas9-derived GDSs for 50 breast cancer cell lines were obtained from the DepMap portal (https://depmap.org/portal/ (accessed on 1 January 2023)) [31,46,47] as log_2_ (TPM + 1) normalized to reconstruct and simulate cell-line-specific GEMs. Expression data were used to reconstruct cell-line-specific GEMs, while GDSs were used as ground truth labels for predicting gene essentiality classification. The 50 cell lines comprised 8 luminal A, 7 luminal B, 10 HER2-positive, and 25 TNBC (Supplementary Table S1).

### 3.2. Reconstruction of Cell-Line-Specific GEMs

Recon 2M.2, a generic human GEM [26] comprising 5825 reactions, 3368 metabolites, and 1682 genes, was used as a reference network for model reconstruction. Cell-line-specific GEMs were constructed by the tINIT algorithm, which integrates RNA-seq data with the reference network to generate a context-specific metabolic model. The reconstruction procedures followed the protocol previously described [32], and implementations are available at https://bitbucket.org/kaistmbel/recon-manager (accessed on 1 January 2023). Expressed genes were ranked, and the top 25% of expressions were assigned positive scores and incorporated into the model reconstruction process under the same conditions validated in the original study [22,48,49]. All cell-line-specific GEM were assessed with 56 metabolic tasks for the tINIT (Supplementary Table S2), and the simulations were performed under the environmental metabolic condition of the Roswell Park Memorial Institute-1640 (RPMI-1640) medium (Supplementary Table S3).

### 3.3. Simulations and Gene Essentiality

MOMA predicts metabolic flux distributions following genetic perturbation by minimizing the Euclidean distance between the perturbed flux vector and the wild-type flux state [50,51]. Unlike flux FBA, which assumes immediate re-optimization after perturbation, MOMA assumes that the perturbed system maintains a flux distribution close to the original metabolic state. For MOMA, tINIT-based cell-line-specific GEMs were used to simulate reaction fluxes for knockout status. MOMA-based gene knockout lethality was defined by a growth rate below 5%, a threshold widely adopted in previous GEM-based gene essentiality studies to represent near-complete growth impairment. GDS values were obtained from DepMap CRISPR-Cas9 knockout screens, as mentioned above, and calculated using the Chronos algorithm [52]. A GDS value close to 1 indicates strong dependency, whereas a value near 0 indicates minimal impact on cell viability. All GEM simulations and operations were implemented using COBRApy [53].

### 3.4. Machine Learning Algorithms

Machine learning models were trained to predict gene knockout lethality using MOMA-derived metabolic flux distribution, with or without genetic perturbation, as input features, while gene essentiality was used as the prediction target. The essentiality label was evaluated using GDSs, with a value of 1 assigned to essential genes (GDS ≥ 0.5) and a value of 0 assigned to non-essential genes (GDS < 0.5).

Four machine learning classifiers were implemented using Scikit-learn library in Python (v.3.6): SVM [54,55], LR [56,57,58], RF [59,60], and NN [61,62]. Hyperparameters of each model were optimized via grid-search cross-validation (Supplementary Table S4). To address the class imbalance between essential and non-essential genes, three sampling strategies were evaluated: (i) the SMOTE, which generates synthetic minority samples using k-nearest neighbors (k = 5); (ii) RandomUnderSampler, which selects the number of non-essential genes to match the number of essential genes; (iii) SMOTE-ENN, which combines SMOTE with edited nearest neighbors to remove borderline noisy samples. Performance was evaluated using mean MCC across 5-fold cross-validation to identify the method with the best performance. To ensure unbiased evaluation of model performance, genes were randomly partitioned into a training and validation set (66.7%) and an independent hold-out test set (33.3%), representing a strict 2:1 split ratio. To ensure strict isolation and prevent data leakage, the test set was completely excluded from all downstream feature selection and model optimization steps. Furthermore, within the 5-fold cross-validation framework, feature selection was performed independently within each fold utilizing only the designated training partition of that specific fold, rather than across the entire training cohort simultaneously. This protocol successfully averted cross-fold information mixing, ensuring that validation fold metrics remained completely untainted before final validation was executed on the sequestered test set, ensuring strict separation of the data used for training, hyperparameter tuning, and model performance. Performance was evaluated based on five metrics: ACC, SPE, SEN, PRE, and MCC:

$$\mathrm{ACC}\;=\;\frac{TP+TN}{TP+FN+FP+TN}$$

$$\mathrm{SPE}=\frac{TN}{TN+FP}$$

$$\mathrm{SEN}=\frac{TP}{TP+FN}$$

$$\mathrm{PRE}=\frac{TP}{TP+FP}$$

$$\mathrm{MCC}=\frac{TP\times TN-FP\times FN}{\sqrt{\left(TP+FP)(TP+FN)(TN+FP)(TN+FN\right)}}$$

where P indicates positive cases, N represents negative cases, T indicates true predictions, and F represents false predictions. Genetic perturbation lethality was predicted for each cell line using gene-knockout lethality prediction models.

As the performance metrics (ACC, SPE, SEN, PRE, and MCC) for the metabolic models across different cell lines did not necessarily follow a normal distribution, the Mann–Whitney-U test (also known as the Wilcoxon rank-sum test) was employed to determine if there were statistically significant differences between the MOMA and ML-based results. This non-parametric approach was selected to ensure robustness against potential outliers and varying distributions within the breast cancer subtypes. A *p*-value of less than 0.05 was considered statistically significant.

To provide a more comprehensive assessment of the effect size beyond simple *p*-values, we performed estimation statistics based on the mean difference between the two approaches. We employed a bootstrapping procedure with resamples (n = 1000) to generate a distribution of the mean difference for each performance metric across all molecular subtypes. For each resample, the data were sampled with replacement to calculate the bootstrap distribution. From this distribution, the 95% bias-corrected and accelerated (BCa) confidence intervals (CIs) were derived.

### 3.5. Synthetic Lethality of Gene Knockouts and Synergy Scores

To identify synthetic lethal gene pairs in TNBC, the trained RF models were applied to predict lethality scores for pairwise gene knockout involving succinate dehydrogenase subunit A (*SDHA*). *SDHA* was selected as the anchor gene based on its mutation in approximately 3% of breast cancer patients [39], making it a clinically relevant candidate for investigating synthetic lethality in TNBC. Synthetic lethality simulations were performed by fixing *SDHA* as the constant component and pairing it with all metabolic genes present in each TNBC cell-line-specific GEM. Across 25 TNBC cell lines, approximately 1200 metabolic genes per cell line were evaluated, resulting in approximately 30,000 double-knockout simulations. Probabilistic lethality (*P_L_*) for each gene *i*, *P_L,i_* was assessed for individual TNBC cell lines using metabolic fluxes derived from cell-line-specific GEMs and GDSs, with prediction of the trained machine learning model. Then, the Bliss independence calculation method, commonly utilized in drug combination analysis, was applied [63].

$$P_{L,c,i}=f_{c}(V_{i})$$

$$I_{Bliss,exp}=1-\prod_{i}^{G}\left(1-P_{i}\right)$$

$$Syn_{L,i,j,exp}=P_{L,i}+P_{L,j}(1-P_{L,i})$$

$$Syn_{L,i,j}=P_{L,i,j}-Syn_{L,i,j,exp}$$

where *f_c_* is the trained machine learning model for cell line *c*, which takes *V_i_*, the reaction flux vector when gene set *i* is perturbed, and predicts *P_L,i_*, the lethality of the flux status. For the Bliss independence, expectation, *I_Bliss,exp_* can be calculated when component *i* is a component of group *G*. Using this, *Syn_L,i,j_*, the synthetic lethality score, can be calculated based on the predictive lethality for the double-knockout status, *P_L,i,j_*, a model result for the double-knockout simulation, and the expectation of *Syn_L,i,j_*, which can also be calculated using *P_L,i_* and *P_L,j_*.

## 4. Conclusions

We applied a metabolic-flux-based machine learning approach to predict gene perturbation lethality. This new method outperformed MOMA in terms of accuracy in predicting gene essentiality. For TNBC, the machine-learning-based approach predicted more rational essential genes, such as the *ENO* family. Additionally, it predicted more synthetic lethal gene pairs for the *SDHA* mutation than the MOMA algorithm. Therefore, this approach captured complex gene interactions and identified a broader range of synthetic lethal genes than did MOMA, offering more comprehensive and rational results. While the present work establishes the framework on 50 breast cancer cell lines, the same MOMA–RF architecture is, in principle, applicable to other cancer lineages catalogued in DepMap; extending and re-benchmarking the pipeline on these lineages is a natural direction for future work. Furthermore, we acknowledge that in vitro cell line models do not fully recapitulate the complex, microenvironmental metabolic characteristics of human tumors in vivo due to culture media adaptations. While the literature indicates that cell line models preserve key cell-autonomous metabolic constraints and core enzyme expressions observed in human cohorts, bridging this translational gap remains crucial. A vital direction for future prospective studies will involve directly integrating patient-specific transcriptomic profiles from clinical cohorts, such as TCGA, to construct personalized genome-scale metabolic models. Direct simulation of genetic perturbations within patient-specific constraints will suppress false-positive rates and significantly enhance the translational fidelity of predicted synthetic lethal gene pairs in clinical anticancer drug development.

## Data Availability

The code and data used and analyzed in this study are available at https://zenodo.org/records/15909194 (accessed on 15 July 2025).

## Supplementary Materials

The following supporting information can be downloaded at: https://www.mdpi.com/article/10.3390/ijms27115059/s1.
