# Supplementary material for: Integrating Genome-Scale Metabolic Modeling with Machine Learning Improves Gene Essentiality Prediction in Triple-Negative Breast Cancer
Source: Int J Mol Sci. 2026 Jun 3;27(11):5059. doi: 10.3390/ijms27115059 (PMC13256420; doi:10.3390/ijms27115059)
Supplement: Supplementary file 1 [file ijms-27-05059-s001.zip › ijms-4273711-supplementary.pdf]

Supplementary Information

Integrating Genome-Scale Metabolic Modeling with Machine Learning Improves Gene Essentiality Prediction in Triple-Negative Breast Cancer

Bo Kyung Kim[1†], Changdai Gu[2,3†], Mohamed El-Agamy Farh[4], Jae Yong Ryu[4,5*]

[1]Artificial Intelligence Laboratory, Oncocross Co., Ltd., 7, Beobwon-ro 11-gil, Songpa-gu, Seoul 05836, Republic of Korea

[2]Department of Artificial Intelligence, School of Computing, Yonsei University, 50 Yonsei-ro, Seodaemun-gu, Seoul 03722, Republic of Korea

[3]Medical Research Center, College of Medicine, Yonsei University, 50 Yonsei-ro, Seodaemun-gu, Seoul 03722, Republic of Korea

[4]AI-Bio Convergence Research Institute, Soongsil University, 369 Sangdo-ro, Dongjak-gu, Seoul 06978, Republic of Korea

[5]School of Systems Biomedical Science, Soongsil University, 369 Sangdo-ro, Dongjak-gu, Seoul 06978, Republic of Korea

*Correspondence: jyryu@ssu.ac.kr

†These authors contributed equally to this work.

Supplementary Figures

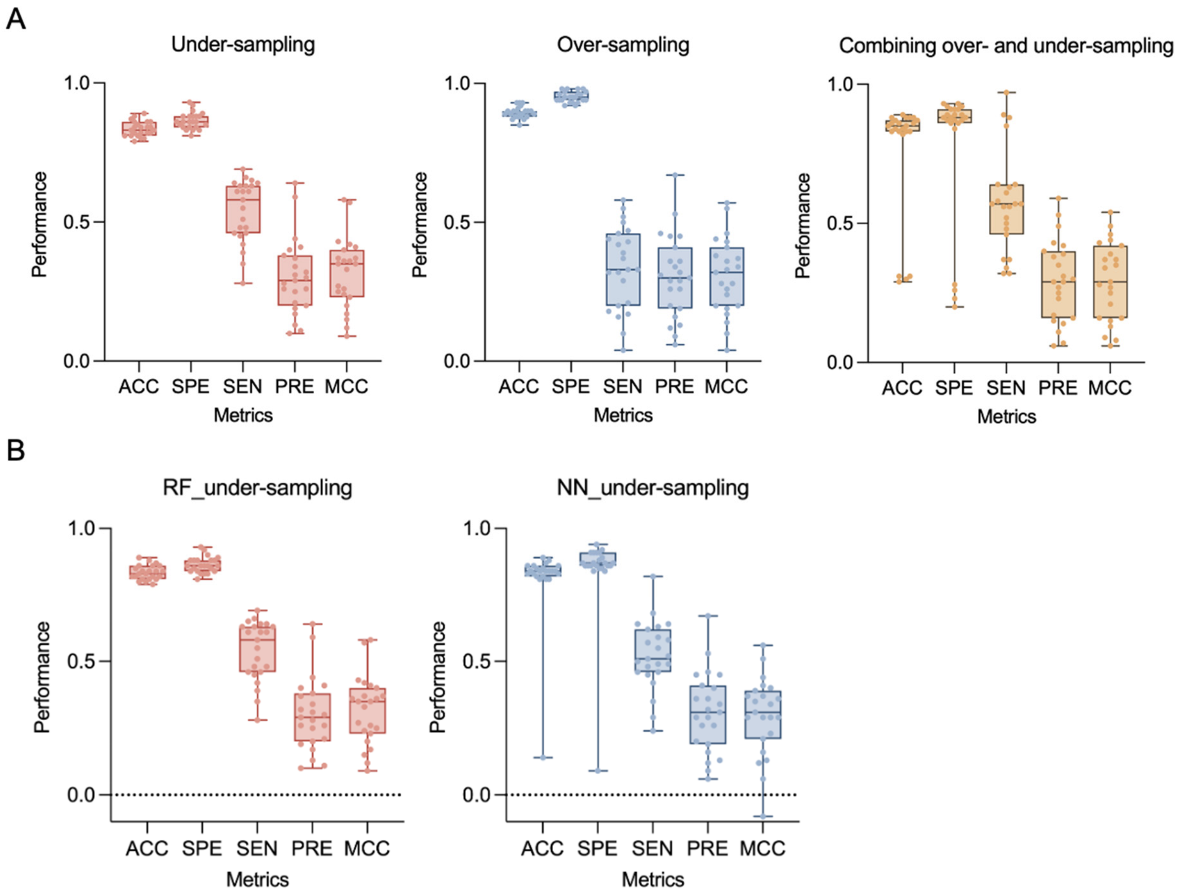

Supplementary Figure S1. Performance evaluation of class imbalance methods for gene essentiality prediction in TNBC cell lines. (A) Comparative evaluation of the efficiency of class imbalance methods to handle data imbalance using the MDA-MB-231 cell line as a representative TNBC cell line. Random under-sampling shows optimal performance. (B) Performance evaluation of random forest and neural network models across all TNBC cell lines. ACC, accuracy; SPE, specificity; SEN, sensitivity; PRE, precision; MCC, Matthews correlation coefficient.

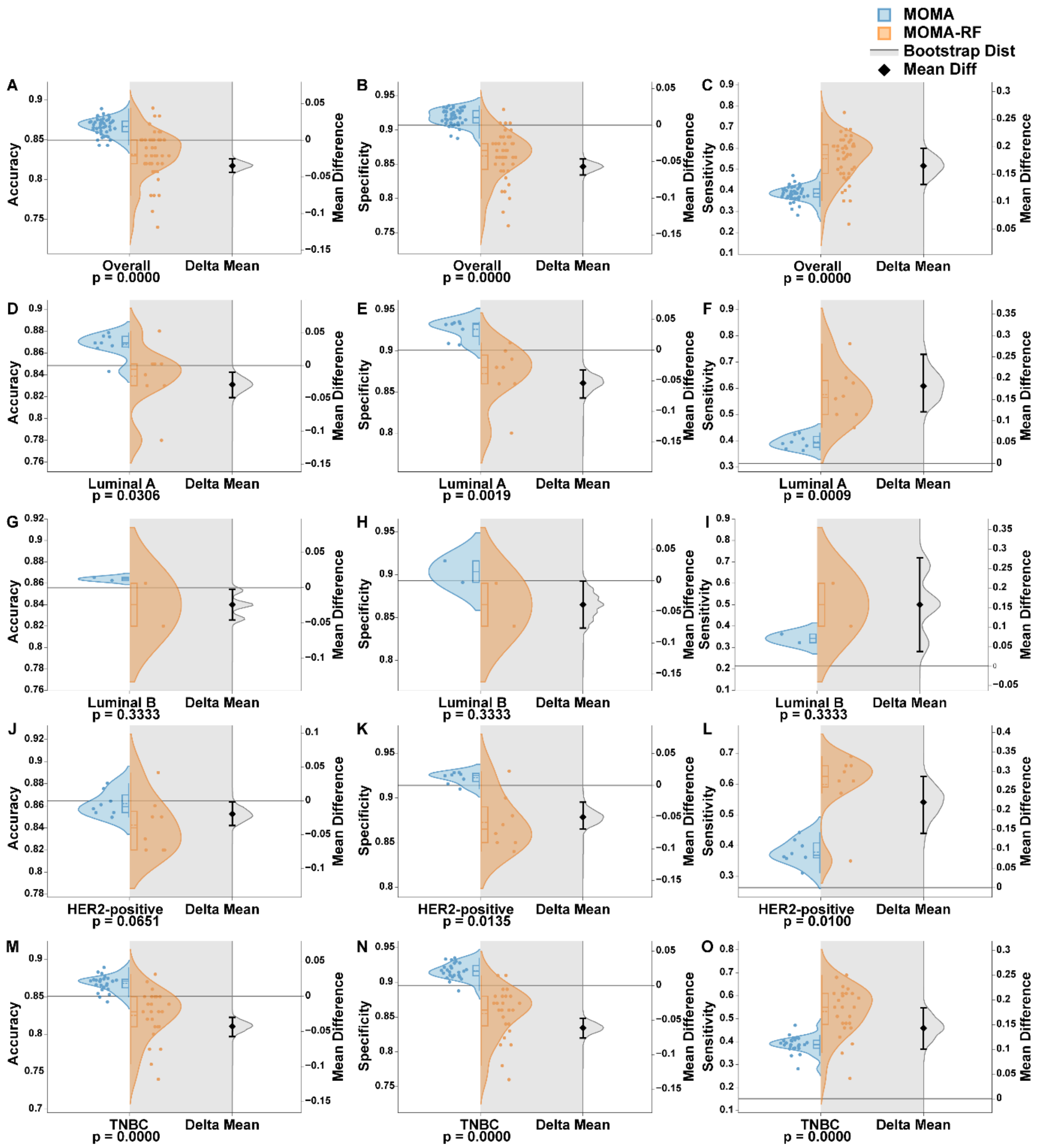

Supplementary Figure S2. Comparison of gene essentiality prediction performance between MOMA and MOMA-RF across breast cancer subtypes. Estimation plots are presented for three metrics: accuracy, specificity, and sensitivity. Generally, MOMA-RF (orange) exhibited lower accuracy and specificity compared to MOMA (blue), but demonstrated a substantial increase in sensitivity. The right axes (grey background) display the Mean Difference (black diamond) between the two models. Shaded grey curves represent the bootstrap distribution of the mean difference, with vertical error bars indicating the 95% confidence intervals (CI). *p*-values derived from the Mann-Whitney U test are provided below each subtype label.

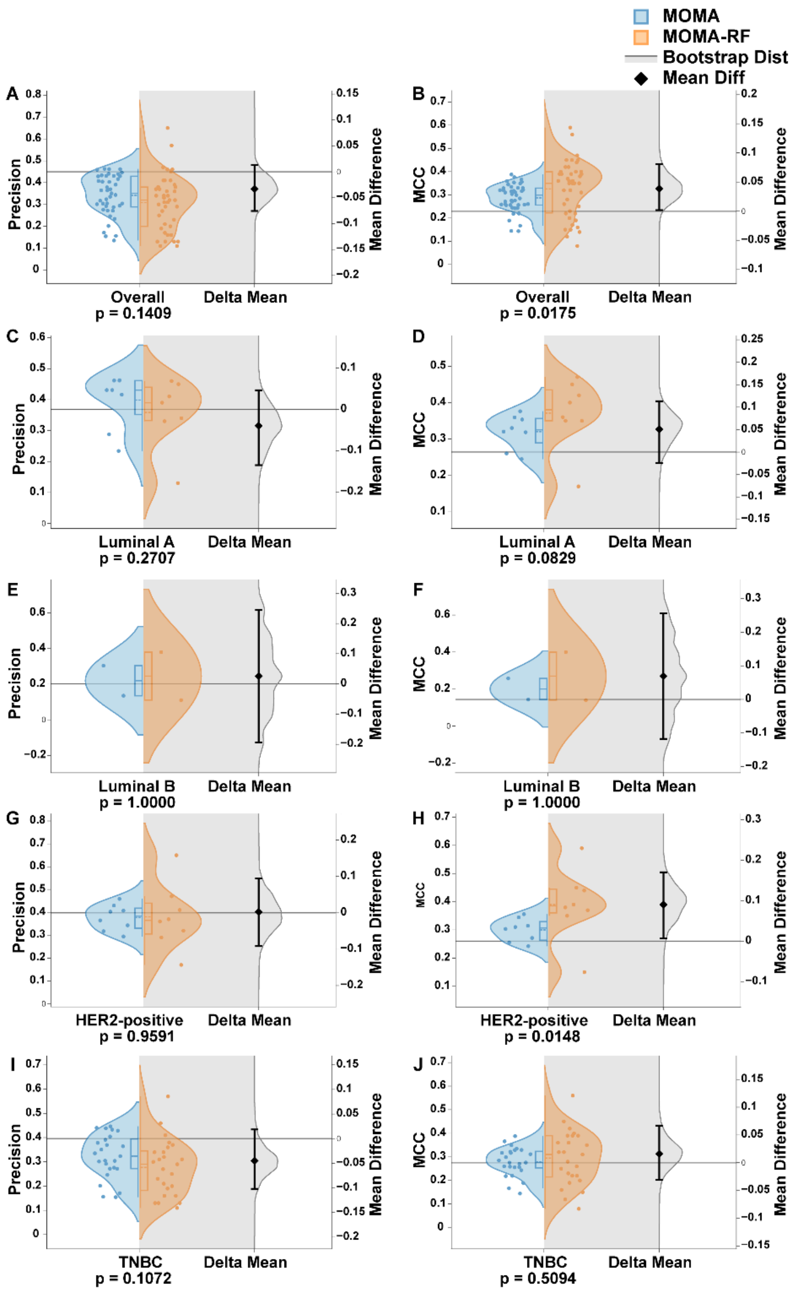

Supplementary Figure S3. Comparison of gene essentiality prediction performance between MOMA and MOMA-RF across breast cancer subtypes. Estimation plots are presented for two metrics: precision and Matthews Correlation Coefficient (MCC). In most cases, MOMA-RF (orange) showed higher MCC values than MOMA (blue), while no significant differences were observed in precision. The right axes (grey background) display the Mean Difference (black diamond) between the two models. Shaded grey curves represent the bootstrap distribution of the mean difference, with vertical error bars indicating the 95% confidence intervals (CI). p-values derived from the Mann-Whitney U test are provided below each subtype label.

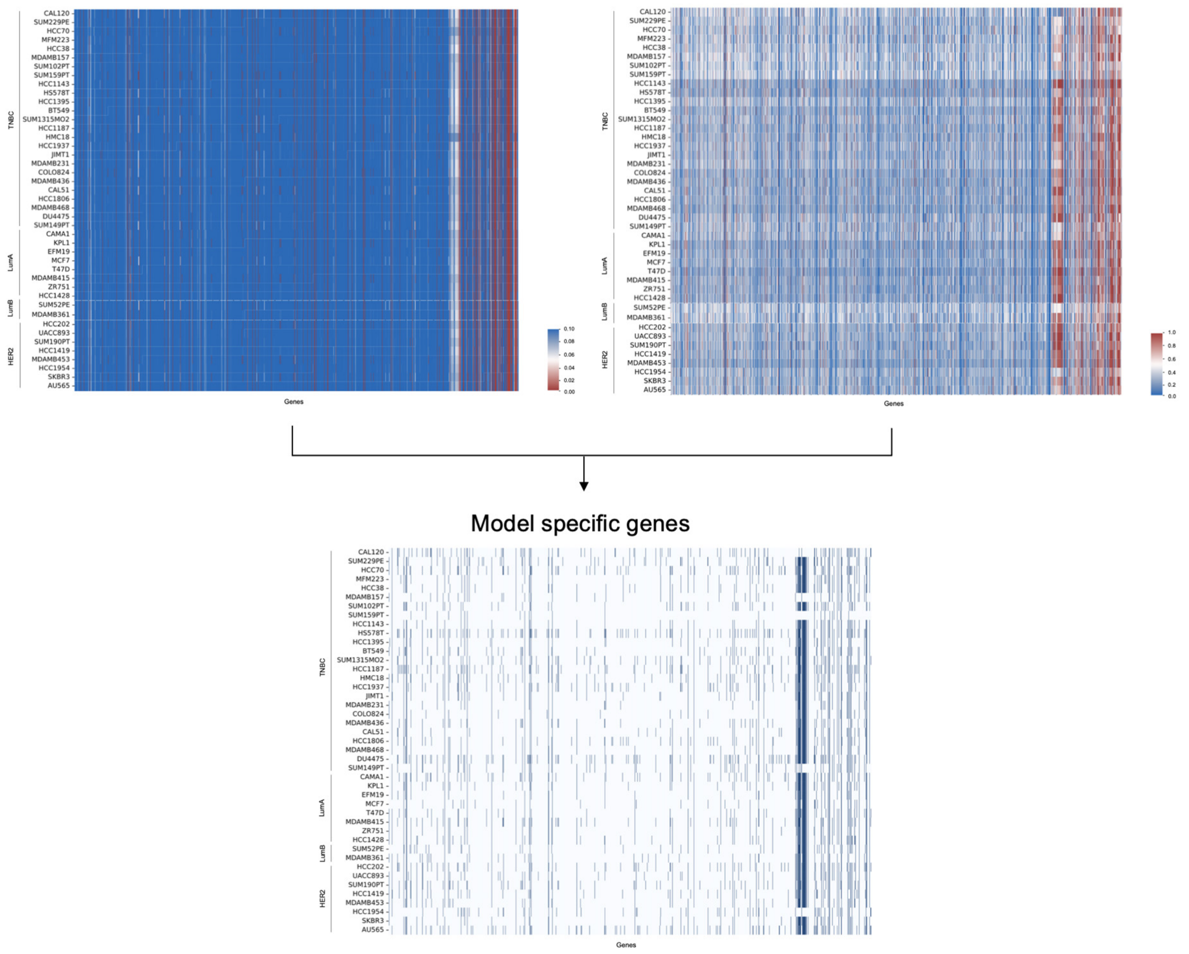

Supplementary Figure S4. Comparative analysis of the gene essentiality prediction accuracies of the minimization of metabolic adjustment (MOMA) and machine learning approaches for various breast cancer subtypes. Heatmaps showing the essentiality patterns of metabolic genes across cell lines of four breast cancer subtypes (luminal A, luminal B, HER2-positive, and TNBC). (Left) MOMA-based predictions, with each value representing the normalized ratio of growth reaction flux (gene knockout growth reaction flux/maximum growth reaction flux) for each cell line; (Right) machine learning model predictions, showing probability values for gene essentiality. Differential analysis (bottom panel) of the genes (blue) predicted as non-essential by MOMA and essential by the machine learning models, identifying 298 novel therapeutic targets. Color intensity indicates the degree of essentiality. Red, essential genes; blue, non-essential genes.

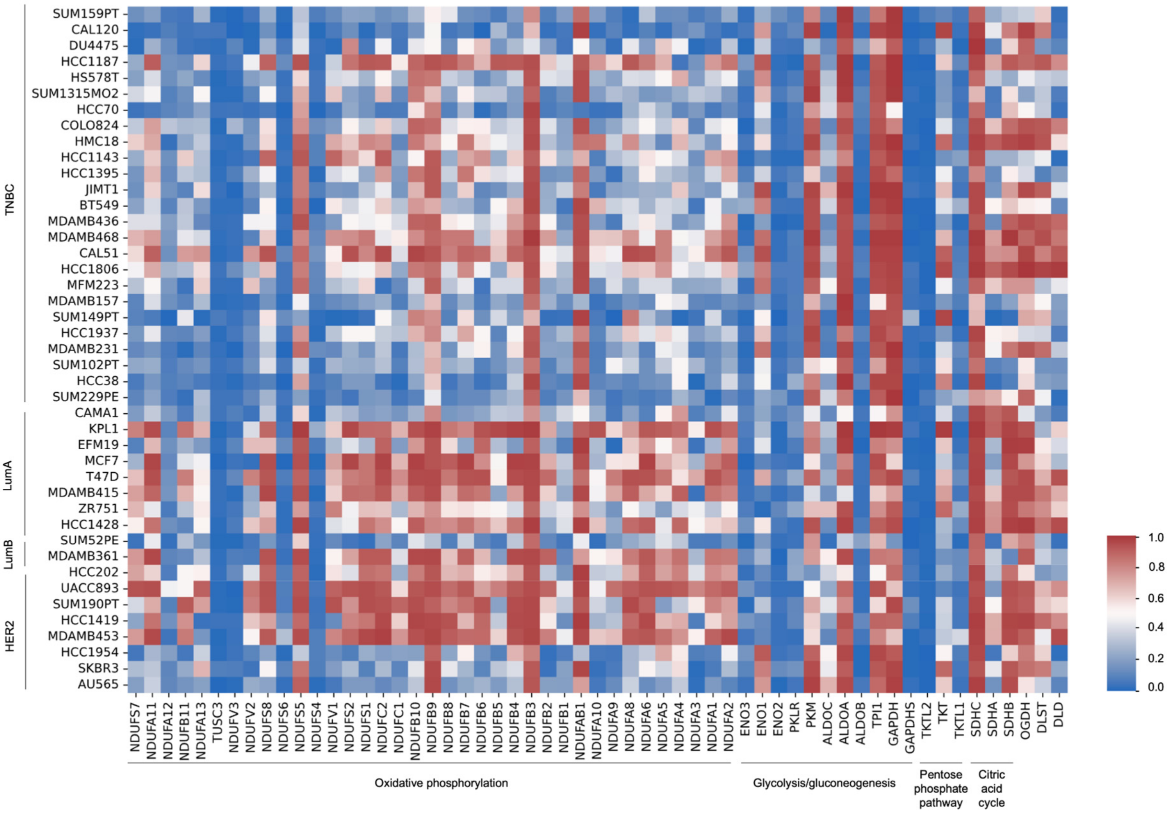

Supplementary Figure S5. Gene dependency score (GDS) analysis of TNBC-specific essential genes across breast cancer subtypes. Heatmap of the GDS patterns for 57 genes predicted as essential by the machine learning model using the TNBC cell lines. These genes were identified from an initial set of 298 candidates that showed differential essentiality between the MOMA and machine learning predictions. Genes are clustered by metabolic pathway, highlighting the pathway-specific essentiality patterns in four breast cancer subtypes (luminal A, luminal B, HER2-positive, and TNBC). Notably, enolase (*ENO*) family genes, particularly *ENO1*, exhibited distinct essentiality in TNBC. Color intensity indicates the degree of essentiality. Red, essential genes; blue, non-essential genes.

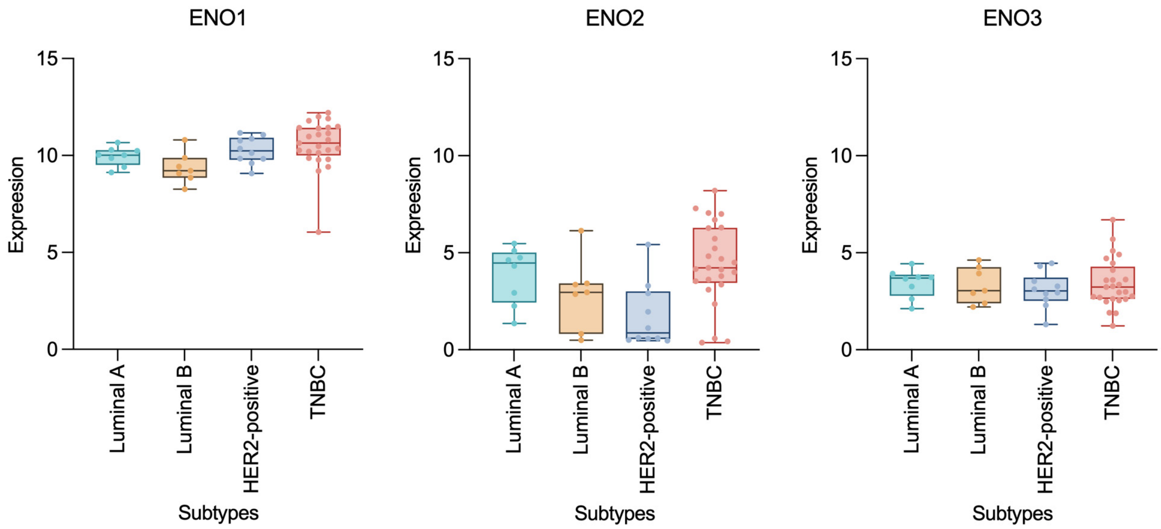

Supplementary Figure S6. Differential expression analysis of *ENO* family genes across breast cancer subtypes. Box plots illustrate the distribution patterns of *ENO1*, *ENO2*, and *ENO3* expression levels across breast cancer cell lines. Each data point represents individual cell line expression values for the respective breast cancer subtype. *ENO1* exhibited higher expression levels than did *ENO2* and *ENO3* in TNBC, correlating with its predicted essentiality in TNBC cell lines. Green, luminal A breast cancer; orange, luminal B breast cancer; blue, HER2-positive breast cancer; red, TNBC.

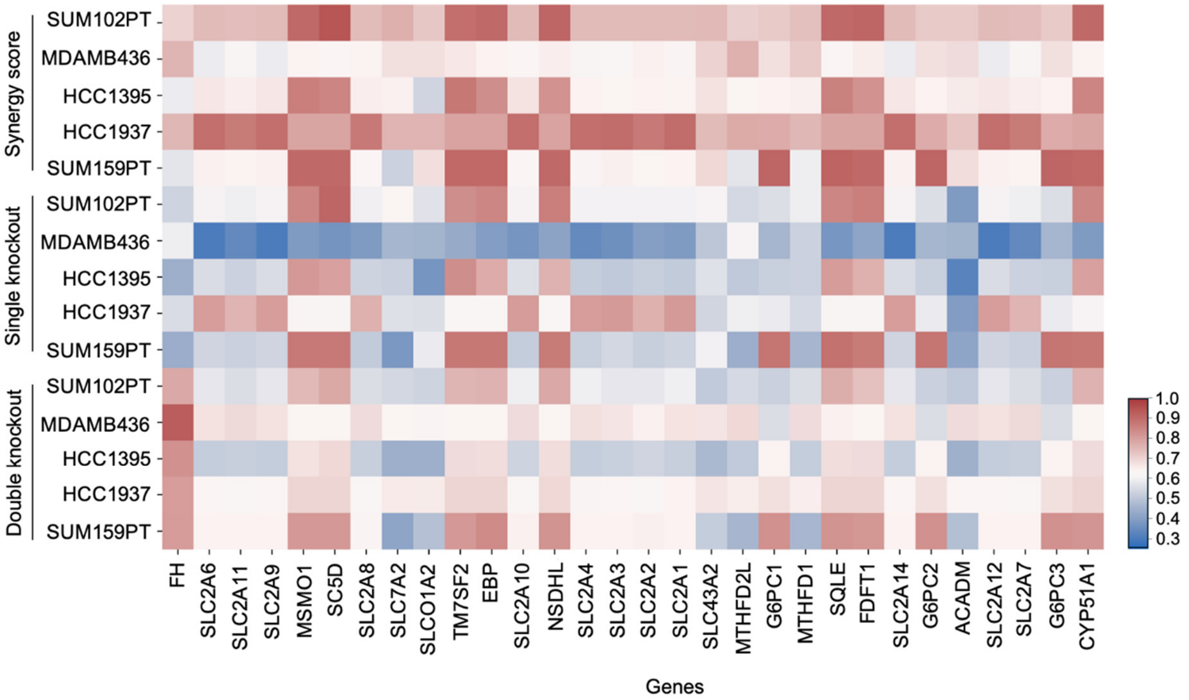

Supplementary Figure S7. Evaluation of the synthetic lethal gene pairs of *SDHA* in TNBC cell lines. Heatmap analysis of 30 predicted synthetic lethal candidate genes in five TNBC cell lines (SUM102PT, MDAMB436, HCC1395, HCC1937, and SUM159PT) with a probabilistic lethality score $P_L < 0.5$ at *SDHA* knockout. The map represents three distinct measurements for each gene: synergy scores, single knockout effects ($P_{L,i}$), and double knockout effects with *SDHA* ($P_{L,SHDA,i}$). The tested candidates, including fumarate hydratase (*FH*), solute carrier (*SLC*)-*2A* family members, and metabolic enzymes, exhibited synthetic lethal relationships beyond those predicted by the MOMA method. Color intensity indicates the degree of essentiality: red, essential genes; blue, non-essential genes.

Supplementary Tables

Supplementary Table S1. Subtypes of breast cancer cell lines in this study.

Subtype (number of cell lines)	Genetic marker status (ER/ PR/ HER2)	Cell lines
luminal A[8]	+ / + / −	CAMA1, EFM19, HCC1428, KPL1, MCF7, MDAMB415, T47D, ZR751
luminal B[7]	+ / + / +	BT474, EFM192A, MDAMB361, SUM44PE, SUM52PE, UACC812, ZR7530
HER2-positive[10]	− / − / +	AU565, HCC1419, HCC1569, HCC1954, HCC202, HCC2218, MDAMB453, SKBR3, SUM190PT, UACC893
TNBC[25]	− / − / −	BT549, CAL120, CAL51, COLO824, DU4475, HCC1143, HCC1187, HCC1395, HCC1806, HCC1937, HCC38, HCC70, HMC18, HS578T, JIMT1, MDAMB157, MDAMB231, MDAMB436, MDAMB468, MFM223, SUM102PT, SUM1315MO2, SUM49PT, SUM159PT, SUM229PE

Supplementary Table S2. Information on 56 metabolic tasks obtained using the Task-Driven Integrative Network Inference for Tissues (tINIT) algorithm.

ID	Description	In	Out	EQU
**Rephosphorylation of nucleoside triphosphates**				
ER	Aerobic rephosphorylation of ATP from glucose	e[s]	H2O[s];CO2[s]	ATP[c] + H2O[c] => ADP[c] + Pi[c]
ER	Aerobic rephosphorylation of GTP	O2[s];glucose[s]	H2O[s];CO2[s]	GTP[c] + H2O[c] => GDP[c] + Pi[c]
ER	Aerobic rephosphorylation of CTP	O2[s];glucose[s]	H2O[s];CO2[s]	CTP[c] + H2O[c] => CDP[c] + Pi[c]
ER	Aerobic rephosphorylation of UTP	O2[s];glucose[s]	H2O[s];CO2[s]	UTP[c] + H2O[c] => UDP[c] + Pi[c]
**De novo synthesis of nucleotides**				
BS	ATP de novo synthesis	O2[s];glucose[s];NH3[s];Pi[s]	H2O[s];CO2[s];ATP[c]	ATP[c] + H2O[c] <=> ADP[c] + Pi[c]
BS	CTP de novo synthesis	O2[s];glucose[s];NH3[s];Pi[s]	H2O[s];CO2[s];CTP[c]	ATP[c] + H2O[c] <=> ADP[c] + Pi[c]
BS	GTP de novo synthesis	O2[s];glucose[s];NH3[s];Pi[s]	H2O[s];CO2[s];GTP[c]	ATP[c] + H2O[c] <=> ADP[c] + Pi[c]
BS	UTP de novo synthesis	O2[s];glucose[s];NH3[s];Pi[s]	H2O[s];CO2[s];UTP[c]	ATP[c] + H2O[c] <=> ADP[c] + Pi[c]
BS	dATP de novo synthesis	O2[s];glucose[s];NH3[s];Pi[s]	H2O[s];CO2[s];dATP[c]	ATP[c] + H2O[c] <=> ADP[c] + Pi[c]
BS	dCTP de novo synthesis	O2[s];glucose[s];NH3[s];Pi[s]	H2O[s];CO2[s];dCTP[c]	ATP[c] + H2O[c] <=> ADP[c] + Pi[c]
BS	dGTP de novo synthesis	O2[s];glucose[s];NH3[s];Pi[s]	H2O[s];CO2[s];dGTP[c]	ATP[c] + H2O[c] <=> ADP[c] + Pi[c]
BS	dTTP de novo synthesis	O2[s];glucose[s];NH3[s];Pi[s]	H2O[s];CO2[s];dTTP[c]	ATP[c] + H2O[c] <=> ADP[c] + Pi[c]
**Uptake of essential amino acids**				
SU	Histidine uptake	histidine[s]	histidine[c]	
SU	Isoleucine uptake	isoleucine[s]	isoleucine[c]	
SU	Leucine uptake	leucine[s]	leucine[c]	
SU	Lysine uptake	lysin[s]	lysin[c]	
SU	Methionine uptake	methionine[s]	methionine[c]	
SU	Phenylalanine uptake	phenylalanine[s]	phenylalanine[c]	
SU	Threonine uptake	threonine[s]	threonine[c]	
SU	Tryptophan uptake	tryptophan[s]	tryptophan[c]	
SU	Valine uptake	valine[s]	valine[c]	
**De novo synthesis of key intermediates**				
IC	Glycerate 3-phosphate de novo synthesis	O2[s];glucose[s];Pi[s]	3-phospho-D-glycerate[c];CO2[s];H2O[s]	
IC	Mitochondrial acetyl-CoA de novo synthesis	O2[s];glucose[s]	CO2[s];H2O[s]	acetyl-CoA[m] => CoA[m]
IC	Mitochondrial AKG de novo synthesis	O2[s];glucose[s]	AKG[m];CO2[s];H2O[s]	
IC	Erythrose 4-phosphate de novo synthesis	O2[s];glucose[s];Pi[s]	erythrose-4-phosphate[c];CO2[s];H2O[s]	
IC	Fructose 6-phosphate de novo synthesis	O2[s];glucose[s];Pi[s]	fructose-6-phosphate[c];CO2[s];H2O[s]	
IC	Glyceraldehyde 3-phosphate de novo synthesis	O2[s];glucose[s];Pi[s]	GAP[c];CO2[s];H2O[s]	
IC	Glucose 6-phosphate de novo synthesis	O2[s];glucose[s];Pi[s]	glucose-6-phosphate[c];CO2[s];H2O[s]	
IC	Mitochondrial oxaloacetate de novo synthesis	O2[s];glucose[s];Pi[s]	OAA[m];CO2[s];H2O[s]	

IC	Phosphoenolpyruvate de novo synthesis	O2[s];glucose[s];Pi[s]	PEP[c];CO2[s];H2O[s]	
IC	Pyruvate de novo synthesis	O2[s];glucose[s];Pi[s]	pyruvate[c];CO2[s];H2O[s]	
IC	Ribose 5-phosphate de novo synthesis	O2[s];glucose[s];Pi[s]	ribose-5-phosphate[c];CO2[s];H2O[s]	
IC	Mitochondrial succinyl-CoA de novo synthesis	O2[s];glucose[s];Pi[s]	CO2[s];H2O[s]	succinyl-CoA[m] => CoA[m]
**De novo synthesis of other compounds**				
BS	Cholesterol de novo synthesis	O2[s];glucose[s]	cholesterol[c]	
**Protein turnover**				
BS	Protein synthesis from AAs	O2[s];glucose[s];NH3[s]; H2O[s]	albumin[c]; CO2[s];H2O[s];H2S[s];urea[s]	
**Electron transport chain and TCA**				
ER	Oxidative phosphorylation	succinate[m];NADH[m]; H+[m];O2[s]	fumarate[m];NAD+[m];H2O[s]	ATP[m] + H2O[m] => ADP[m] + Pi[m]
ER	Oxidative decarboxylation	pyruvate[m];NAD+[m];CoA[m]	acetyl-CoA[m];NADH[m];H+[m];CO2[s]	
ER	Krebs cycle NADH	acetyl-CoA[m];GDP[m];ubiquinone[m]; NAD+[m];Pi[m];H2O[s]	CoA[m];ubiquinol[m];GTP[m];NADH[m];CO2[s];H+[c];H+[m]	
ER	Ubiquinol-to-proton	ubiquinol[m];O2[s];H+[m]	ubiquinone[m];H2O[s];H+[c]	
ER	Ubiquinol-to-ATP	ubiquinol[m];O2[s];H+[m]	ubiquinone[m];H2O[s];H+[m];H+[c]	ATP[m] + H2O[m] => ADP[m] + Pi[m]
**Beta oxidation of fatty acids**				
SU	Beta oxidation of saturated FA	stearate[s];O2[s]	H2O[s];CO2[s]	ATP[c] + H2O[c] => ADP[c] + Pi[c]
SU	Beta oxidation of long-chain FA	12,15,18,21-tetracosatetraenoic acid[s];O2[s]	H2O[s];CO2[s]	
SU	Beta oxidation of odd-chain FA	margaric acid[s];O2[s]	H2O[s];CO2[s]	ATP[c] + H2O[c] => ADP[c] + Pi[c]
SU	Beta oxidation of unsaturated fatty acid (n-9)	ximenic acid[s];O2[s]	H2O[s];CO2[s]	
SU	Beta oxidation of unsaturated fatty acid (n-6)	linoleate[s];O2[s]	H2O[s];CO2[s]	ATP[c] + H2O[c] => ADP[c] + Pi[c]
SU	Uptake and beta oxidation of all NEFAs	NEFA blood pool in[x];O2[s]	H2O[s];CO2[s]	ATP[c] + H2O[c] => ADP[c] + Pi[c]
**De novo synthesis of phospholipids**				
SU	Choline uptake	choline[s]	choline[c]	
SU	Inositol uptake	inositol[s]	inositol[c]	
BS	Phosphatidylcholine de novo synthesis	choline[s];glucose[s];O2[s];NEFA blood pool in[s];Pi[s]	PC-LD pool[c];H2O[s];CO2[s]	ATP[c] + H2O[c] <=> ADP[c] + Pi[c]
BS	Phosphatidylethanolamine de novo synthesis	ethanolamine[s];glucose[s];O2[s];NEFA blood pool in[s];Pi[s]	PE-LD pool[c];H2O[s];CO2[s]	ATP[c] + H2O[c] <=> ADP[c] + Pi[c]
BS	Phosphatidylserine de novo synthesis	serine[s];glucose[s];O2[s];NEFA blood pool in[s];Pi[s]	PS-LD pool[c];H2O[s];CO2[s]	ATP[c] + H2O[c] <=> ADP[c] + Pi[c]
BS	Phosphatidylinositol de novo synthesis	inositol[s];glucose[s];O2[s];NEFA blood pool in[s];Pi[s]	PI pool[c];H2O[s];CO2[s]	ATP[c] + H2O[c] <=> ADP[c] + Pi[c]
**Vitamins and co-factors**				
BS	Thiamin phosphorylation to TPP	thiamin[s];Pi[s];O2[s];H2O[s]	thiamin-PP[c];H2O[s]	ATP[c] + H2O[c] <=> ADP[c] + Pi[c]
BS	Coenzyme A synthesis from pantothenate	pantothenate[s];cysteine[s];glucose[s];O2[s];H2O[s];Pi[s];NH3[s]	CoA[c]; H2O[s];CO2[s]	ATP[c] + H2O[c] <=> ADP[c] + Pi[c]
BS	FAD synthesis from riboflavin	O2[s];glucose[s];NH3[s]; Pi[s];riboflavin[s]	FAD[c];H2O[s];CO2[s]	

BS	Heme biosynthesis	O2[s];NH3[s];glucose[s];<br>Fe2+[s]	heme[c];H2O[s];CO2[s]	ATP[c] + H2O[c]<br><=> ADP[c] + Pi[c]

ER, energy and redox; IC, internal conversion; SU, substrate utilization; BS, biosynthesis of products.

Supplementary Table S3. Information on constraints for the Roswell Park Memorial Institute (RPMI)-1640 medium.

Medium	Reaction ID	Lower bound	Upper bound
	EX_ala_L_LPAREN_e_RPAREN_	−0.05	1000
	EX_arg_L_LPAREN_e_RPAREN_	−0.05	1000
	EX_asn_L_LPAREN_e_RPAREN_	−0.05	1000
	EX_asp_L_LPAREN_e_RPAREN_	−0.05	1000
	EX_btn_LPAREN_e_RPAREN_	−0.005	1000
	EX_ca2_LPAREN_e_RPAREN_	−1000	1000
	EX_chol_LPAREN_e_RPAREN_	−0.005	1000
	EX_cl_LPAREN_e_RPAREN_	−1000	1000
	EX_co2_LPAREN_e_RPAREN_	−1000	1000
	EX_cys_L_LPAREN_e_RPAREN_	−0.05	1000
	EX_fe2_LPAREN_e_RPAREN_	−1000	1000
	EX_fe3_LPAREN_e_RPAREN_	−1000	1000
	EX_fol_LPAREN_e_RPAREN_	−0.005	1000
	EX_glc_LPAREN_e_RPAREN_	−5	1000
	EX_gln_L_LPAREN_e_RPAREN_	−0.5	1000
	EX_glu_L_LPAREN_e_RPAREN_	−0.05	1000
RPMI-1640 medium	EX_gly_LPAREN_e_RPAREN_	−0.05	1000
	EX_gthrd_LPAREN_e_RPAREN_	−0.05	1000
	EX_h_LPAREN_e_RPAREN_	−1000	1000
	EX_h2o_LPAREN_e_RPAREN_	−1000	1000
	EX_his_L_LPAREN_e_RPAREN_	−0.05	1000
	EX_ile_L_LPAREN_e_RPAREN_	−0.05	1000
	EX_inost_LPAREN_e_RPAREN_	−0.005	1000
	EX_k_LPAREN_e_RPAREN_	−1000	1000
	EX_leu_L_LPAREN_e_RPAREN_	−0.05	1000
	EX_lys_L_LPAREN_e_RPAREN_	−0.05	1000
	EX_met_L_LPAREN_e_RPAREN_	−0.05	1000
	EX_na1_LPAREN_e_RPAREN_	−1000	1000
	EX_ncam_LPAREN_e_RPAREN_	−0.005	1000
	EX_nh4_LPAREN_e_RPAREN_	−1000	1000
	EX_o2_LPAREN_e_RPAREN_	−1000	1000
	EX_phe_L_LPAREN_e_RPAREN_	−0.05	1000
	EX_pi_LPAREN_e_RPAREN_	−1000	1000
	EX_pnto_R_LPAREN_e_RPAREN_	−0.005	1000

EX_pro_L_LPAREN_e_RPAREN_	−0.05	1000
EX_pydx_LPAREN_e_RPAREN_	−0.005	1000
EX_ribflv_LPAREN_e_RPAREN_	−0.005	1000
EX_ser_L_LPAREN_e_RPAREN_	−0.05	1000
EX_thm_LPAREN_e_RPAREN_	−0.005	1000
EX_thr_L_LPAREN_e_RPAREN_	−0.05	1000
EX_trp_L_LPAREN_e_RPAREN_	−0.05	1000
EX_tyr_L_LPAREN_e_RPAREN_	−0.05	1000
EX_val_L_LPAREN_e_RPAREN_	−0.05	1000
EX_zn2_LPAREN_e_RPAREN_	−1000	1000

Supplementary Table S4. Hyperparameter candidates of machine learning models for optimization.

Model type	Hyperparameter types	Model
Support vector machine (SVM)	C (regularization)	0.1; 0.5; 1; 10; 100
	gamma	“auto”
	kernel	RBF
Logistic regression (LR)	C (regularization strength)	10^{-4}; 10^{-3}; 10^{-2}; 10^{-1}; 0; 10^{1}; 10^{2}; 10^{3}; 10^{4}
	penalty	L2; None
Random forest (RF)	n_estimator	100; 200; 300
	max_depths	10; 20; 30
	min_samples	2; 5; 10
Neural network (NN)	hidden_layer_sizes	(50,); [100]; [50]; [100]
	activation	“relu”
	optimizer	adam
	learning_rate_init	0.01

Supplementary Table S5. Statistical comparison of MOMA and MOMA-RF gene essentiality prediction performance of four breast cancer subtypes (Mann-Whitney U test *p*).

Breast cancer type	Statistics	Accuracy	Specificity	Sensitivity	Precision	MCC
Luminal A	Mann-Whitney U test p	0.0306	0.0019	0.0009	0.2707	0.0829
Luminal A	95% CI	0.0406	0.0434	0.1332	0.1825	0.1347
Luminal A	CI_high*	-0.0103	-0.0329	0.2518	0.0480	0.1145
Luminal A	CI_low**	-0.0509	-0.0763	0.1186	-0.1345	-0.0203
Luminal A	MOMA std	0.0103	0.0108	0.0230	0.0820	0.0435
Luminal A	MOMA-RF std	0.0267	0.0319	0.0945	0.1040	0.0871
Luminal B	Mann-Whitney U test p	0.3333	0.3333	0.3333	1.0000	1.0000
Luminal B	95% CI	0.0429	0.0751	0.2403	0.4386	0.3741
Luminal B	CI_high*	-0.0026	-0.0011	0.2774	0.2449	0.2564
Luminal B	CI_low**	-0.0455	-0.0761	0.0372	-0.1937	-0.1178
Luminal B	MOMA std	0.0015	0.0125	0.0201	0.0843	0.0571
Luminal B	MOMA-RF std	0.0200	0.0250	0.1000	0.1350	0.1300
HER2-positive	Mann-Whitney U test p	0.0651	0.0135	0.0100	0.9591	0.0148
HER2-positive	95% CI	0.0337	0.0410	0.1404	0.1987	0.1568
HER2-positive	CI_high*	-0.0009	-0.0277	0.2841	0.1064	0.1656
HER2-positive	CI_low**	-0.0346	-0.0686	0.1437	-0.0922	0.0088
HER2-positive	MOMA std	0.0102	0.0062	0.0382	0.0533	0.0381
HER2-positive	MOMA-RF std	0.0233	0.0282	0.1003	0.1314	0.1150
TNBC	Mann-Whitney U test p	1.02E-09	1.78E-13	4.10E-11	0.1409	0.0175
TNBC	95% CI	0.0188	0.0213	0.0661	0.0903	0.0783
TNBC	CI_high*	-0.0262	-0.0462	0.1974	0.0131	0.0749
TNBC	CI_low**	-0.0450	-0.0675	0.1313	-0.0772	-0.0034
TNBC	MOMA std	0.0104	0.0116	0.0344	0.0915	0.0586
TNBC	MOMA-RF std	0.0306	0.0347	0.1061	0.1252	0.1177
Overall	Mann-Whitney U test p	0.0000	0.0000	0.0000	0.1409	0.0175
Overall	95% CI	0.0201	0.0198	0.0630	0.0934	0.0799
Overall	CI_high	-0.0248	-0.0474	0.1971	0.0136	0.0771
Overall	CI_low	-0.0449	-0.0672	0.1341	-0.0797	-0.0028
Overall	MOMA std	0.0104	0.0116	0.0344	0.0915	0.0586
Overall	MOMA-RF std	0.0306	0.0347	0.1061	0.1252	0.1177

*CI_high: The 97.5th percentile of the bootstrap distribution.

**CI_low: The 2.5th percentile of the bootstrap distribution.

Supplementary Table S6. Metabolic reactions and corresponding genes for the Figure 4C.

Reaction ID	Reaction name	Genes*
2OXOADOXm	2-Oxoadipate:lipoamde 2-oxidoreductase(decarboxylating and acceptor-succinylating) (mitochondria)	DLD (dihydrolipoamide dehydrogenase); DLST (dihydrolipoamide S-succinyltransferase); OGDH (oxoglutarate dehydrogenase); PDHX (pyruvate dehydrogenase complex component X)
AKGDm	2-oxoglutarate dehydrogenase	DLD (dihydrolipoamide dehydrogenase); DLST (dihydrolipoamide S-succinyltransferase); OGDH (oxoglutarate dehydrogenase); PDHX (pyruvate dehydrogenase complex component X)
ENO	enolase	ENO1 (enolase 1); ENO2 (enolase 2); ENO3 (enolase 3)
FBA	fructose-bisphosphate aldolase	ALDOA (aldolase, fructose-bisphosphate A); ALDOB (aldolase, fructose-bisphosphate B); ALDOC (aldolase, fructose-bisphosphate C)
FBA2	D-Fructose 1-phosphate D-glyceraldehyde-3-phosphate-lyase	ALDOA (aldolase, fructose-bisphosphate A); ALDOB (aldolase, fructose-bisphosphate B); ALDOC (aldolase, fructose-bisphosphate C)
GAPD	glyceraldehyde-3-phosphate dehydrogenase	GAPDH (glyceraldehyde-3-phosphate dehydrogenase); GAPDHS (glyceraldehyde-3-phosphate dehydrogenase, spermatogenic)
GCC2am	glycine-cleavage complex (lipoamide), mitochondrial	AMT (aminomethyltransferase); DLD (dihydrolipoamide dehydrogenase); GCSH (glycine cleavage system protein H); GLDC (glycine decarboxylase)
GCC2bim	glycine-cleavage system (lipoamide) irreversible, mitochondrial	AMT (aminomethyltransferase); DLD (dihydrolipoamide dehydrogenase); GCSH (glycine cleavage system protein H); GLDC (glycine decarboxylase)
GCC2cm	glycine-cleavage complex (lipoamide), mitochondrial	AMT (aminomethyltransferase); DLD (dihydrolipoamide dehydrogenase); GCSH (glycine cleavage system protein H); GLDC (glycine decarboxylase)
GCCbim	glycine-cleavage complex (lipoylprotein) irreversible, mitochondrial	AMT (aminomethyltransferase); DLD (dihydrolipoamide dehydrogenase); GCSH (glycine cleavage system protein H); GLDC (glycine decarboxylase)
GCCcm	glycine-cleavage complex (lipoylprotein), mitochondrial	AMT (aminomethyltransferase); DLD (dihydrolipoamide dehydrogenase); GCSH (glycine cleavage system protein H); GLDC (glycine decarboxylase)
NADH2_u10m	NADH dehydrogenase, mitochondrial	COXFA4 (cytochrome c oxidase associated subunit FA4); MT-ND1 (mitochondrially encoded NADH:ubiquinone oxidoreductase core subunit 1); MT-ND2 (mitochondrially encoded NADH:ubiquinone oxidoreductase core subunit 2); MT-ND3 (mitochondrially encoded NADH:ubiquinone oxidoreductase core subunit 3); MT-ND4 (mitochondrially encoded NADH:ubiquinone oxidoreductase core subunit 4); MT-ND4L (mitochondrially encoded NADH:ubiquinone oxidoreductase core subunit 4L); MT-ND5 (mitochondrially encoded NADH:ubiquinone oxidoreductase core subunit 5); MT-ND6 (mitochondrially encoded NADH:ubiquinone oxidoreductase core subunit 6); NDUFA1 (NADH:ubiquinone oxidoreductase subunit A1); NDUFA10 (NADH:ubiquinone oxidoreductase subunit A10);

		NDUFA11 (NADH:ubiquinone oxidoreductase subunit A11); NDUFA12 (NADH:ubiquinone oxidoreductase subunit A12); NDUFA13 (NADH:ubiquinone oxidoreductase subunit A13); NDUFA2 (NADH:ubiquinone oxidoreductase subunit A2); NDUFA3 (NADH:ubiquinone oxidoreductase subunit A3); NDUFA5 (NADH:ubiquinone oxidoreductase subunit A5); NDUFA6 (NADH:ubiquinone oxidoreductase subunit A6); NDUFA7 (NADH:ubiquinone oxidoreductase subunit A7); NDUFA8 (NADH:ubiquinone oxidoreductase subunit A8); NDUFA9 (NADH:ubiquinone oxidoreductase subunit A9); NDUFAB1 (NADH:ubiquinone oxidoreductase subunit AB1); NDUFB1 (NADH:ubiquinone oxidoreductase subunit B1); NDUFB10 (NADH:ubiquinone oxidoreductase subunit B10); NDUFB11 (NADH:ubiquinone oxidoreductase subunit B11); NDUFB2 (NADH:ubiquinone oxidoreductase subunit B2); NDUFB3 (NADH:ubiquinone oxidoreductase subunit B3); NDUFB4 (NADH:ubiquinone oxidoreductase subunit B4); NDUFB5 (NADH:ubiquinone oxidoreductase subunit B5); NDUFB6 (NADH:ubiquinone oxidoreductase subunit B6); NDUFB7 (NADH:ubiquinone oxidoreductase subunit B7); NDUFB8 (NADH:ubiquinone oxidoreductase subunit B8); NDUFB9 (NADH:ubiquinone oxidoreductase subunit B9); NDUFC1 (NADH:ubiquinone oxidoreductase subunit C1); NDUFC2 (NADH:ubiquinone oxidoreductase subunit C2); NDUFS1 (NADH:ubiquinone oxidoreductase core subunit S1); NDUFS2 (NADH:ubiquinone oxidoreductase core subunit S2); NDUFS3 (NADH:ubiquinone oxidoreductase core subunit S3); NDUFS4 (NADH:ubiquinone oxidoreductase subunit S4); NDUFS5 (NADH:ubiquinone oxidoreductase subunit S5); NDUFS6 (NADH:ubiquinone oxidoreductase subunit S6); NDUFS7 (NADH:ubiquinone oxidoreductase core subunit S7); NDUFS8 (NADH:ubiquinone oxidoreductase core subunit S8); NDUFV1 (NADH:ubiquinone oxidoreductase core subunit V1); NDUFV2 (NADH:ubiquinone oxidoreductase core subunit V2); NDUFV3 (NADH:ubiquinone oxidoreductase subunit V3); TUSC3 (tumor suppressor candidate 3)
OIVD1m	2-oxoisovalerate dehydrogenase (acylating; 4-methyl-2-oxopentaoate), mitochondrial	BCKDHA (branched chain keto acid dehydrogenase E1 subunit alpha); BCKDHB (branched chain keto acid dehydrogenase E1 subunit beta); DBT (dihydrolipoamide branched chain transacylase E2); DLD (dihydrolipoamide dehydrogenase)
OIVD2m	2-oxoisovalerate dehydrogenase (acylating; 3-methyl-2-oxobutanoate), mitochondrial	BCKDHA (branched chain keto acid dehydrogenase E1 subunit alpha); BCKDHB (branched chain keto acid dehydrogenase E1 subunit beta); DBT (dihydrolipoamide branched chain transacylase E2); DLD (dihydrolipoamide dehydrogenase)
OIVD3m	2-oxoisovalerate dehydrogenase (acylating; 3-methyl-2-oxopentanoate), mitochondrial	BCKDHA (branched chain keto acid dehydrogenase E1 subunit alpha); BCKDHB (branched chain keto acid dehydrogenase E1 subunit beta); DBT (dihydrolipoamide branched chain transacylase E2); DLD (dihydrolipoamide dehydrogenase)
PDHm	pyruvate dehydrogenase	DLAT (dihydrolipoamide S-acetyltransferase); DLD

		(dihydrolipoamide dehydrogenase); PDHA1 (pyruvate dehydrogenase E1 subunit alpha 1); PDHA2 (pyruvate dehydrogenase E1 subunit alpha 2); PDHB (pyruvate dehydrogenase E1 subunit beta); PDHX (pyruvate dehydrogenase complex component X)
PYK	pyruvate kinase	PKLR (pyruvate kinase L/R); PKM (pyruvate kinase M1/2)
SUCD1m	succinate dehydrogenase	SDHA (succinate dehydrogenase complex flavoprotein subunit A); SDHB (succinate dehydrogenase complex iron sulfur subunit B); SDHC (succinate dehydrogenase complex subunit C); SDHD (succinate dehydrogenase complex subunit D)
TKT1	transketolase	TKT (transketolase); TKTL1 (transketolase like 1); TKTL2 (transketolase like 2)
TKT2	transketolase	TKT (transketolase); TKTL1 (transketolase like 1); TKTL2 (transketolase like 2)
TPI	triose-phosphate isomerase	TPI1 (triosephosphate isomerase 1)
r0163	2-oxoglutarate dehydrogenase E1 component Citrate cycle (TCA cycle) EC:1.2.4.2	OGDH (oxoglutarate dehydrogenase)
r0384	2-oxoglutarate:[dihydrolipoyllysine-residue succinyltransferase]-lipoyllysine 2-oxidoreductase (decarboxylating, acceptor-succinylating) EC:1.2.4.2	OGDH (oxoglutarate dehydrogenase)
r0407	Sedoheptulose 1,7-bisphosphate D-glyceraldehyde-3-phosphate-lyase Carbon fixation EC:4.1.2.13	ALDOA (aldolase, fructose-bisphosphate A); ALDOB (aldolase, fructose-bisphosphate B); ALDOC (aldolase, fructose-bisphosphate C)
r0451	2-Oxoadipate:lipoamde 2-oxidoreductase(decarboxylating and acceptor-succinylating) Lysine degradation EC:1.2.4.2	OGDH (oxoglutarate dehydrogenase)
r0556	succinyl-CoA:enzyme N6-(dihydrolipoyl)lysine S-succinyltransferase Citrate cycle (TCA cycle) EC:2.3.1.61	DLST (dihydrolipoamide S-succinyltransferase)
r0557	Glutaryl-CoA:dihydrolipoamide S-succinyltransferase Lysine degradation EC:2.3.1.61	DLST (dihydrolipoamide S-succinyltransferase)
r0620	2-oxoglutarate dehydrogenase E1 component Citrate cycle (TCA cycle) EC:1.2.4.2	OGDH (oxoglutarate dehydrogenase)
r1154	EC:1.2.7.2	BCKDHA (branched chain keto acid dehydrogenase E1 subunit alpha); BCKDHB (branched chain keto acid dehydrogenase E1 subunit beta); DBT (dihydrolipoamide branched chain transacylase E2); DLD (dihydrolipoamide dehydrogenase)

*The genes of each corresponding reaction are listed based on the general Recon2M.2 model, and gene IDs and names were identified based on Entrez and HGNC databases.
